# Chemical Structure and Biological Activity of Humic Substances Define Their Role as Plant Growth Promoters

**DOI:** 10.3390/molecules26082256

**Published:** 2021-04-13

**Authors:** Serenella Nardi, Michela Schiavon, Ornella Francioso

**Affiliations:** 1 Department of Agronomy, Food, Natural Resources, Animals and Environment, Università degli Studi di Padova, V.le dell’Università 16, Legnaro, 35020 Padova, Italy; serenella.nardi@unipd.it; 2 Department of di of Agricultural, Forest and Food Sciences (DISAFA), University of Turin, Largo Paolo Braccini 2 (già Via Leonardo da Vinci, 44), 10095 Grugliasco, Italy; 3 Department of Agricultural and Food Sciences, University of Bologna, Viale G. Fanin, 40, 40127 Bologna, Italy; ornella.francioso@unibo.it

**Keywords:** humic substances, hydrophobicity, hydrophily, growth promoters, hormone-like activity, auxin, nutrition, biological activity

## Abstract

Humic substances (HS) are dominant components of soil organic matter and are recognized as natural, effective growth promoters to be used in sustainable agriculture. In recent years, many efforts have been made to get insights on the relationship between HS chemical structure and their biological activity in plants using combinatory approaches. Relevant results highlight the existence of key functional groups in HS that might trigger positive local and systemic physiological responses via a complex network of hormone-like signaling pathways. The biological activity of HS finely relies on their dosage, origin, molecular size, degree of hydrophobicity and aromaticity, and spatial distribution of hydrophilic and hydrophobic domains. The molecular size of HS also impacts their mode of action in plants, as low molecular size HS can enter the root cells and directly elicit intracellular signals, while high molecular size HS bind to external cell receptors to induce molecular responses. Main targets of HS in plants are nutrient transporters, plasma membrane H^+^-ATPases, hormone routes, genes/enzymes involved in nitrogen assimilation, cell division, and development. This review aims to give a detailed survey of the mechanisms associated to the growth regulatory functions of HS in view of their use in sustainable technologies.

## 1. Introduction

For many years, soil scientists have endeavored to define the chemical features and the molecular structure of humic substances (HS), and discover how they can modify the growth and development of plants. A few studies came out with the hypothesis that HS might act in plants through two distinct mechanisms, of which one is indirect and achieved via amelioration of soil chemical, physical and biological properties, while the other implies a more direct effect of HS active components on the regulation of growth processes, nutrient transport systems, and primary and secondary metabolism [1,2].

The biological activity of HS in soil and plants, which is responsible for plant growth promotion, becomes relevant in the context of sustainable agriculture that claims for solutions to address major issues of environmental pollution and economic costs related to fertilizer inputs, while preventing crop yield and quality trade-off. The use of nitrogen-based fertilizers is one of the most energy consuming processes in agricultural practices and its burst is associated to detrimental environmental consequences and significant releases of reactive N species (except N_2_) in the atmosphere. Because only a limited amount of nutrients in fertilizers can be promptly used by plants (e.g., only 30–50% of applied N fertilizers), attention is paid to low-impact agriculture approaches aimed to increase plant nutrient use acquisition and efficiency.

Among these strategies, the use of biostimulants is relevant, of which HS category is part [1,2,3]. Biostimulants by definition are substances that promote plant growth, nutrition and metabolism through modes of action that are challenging to decipher, but definitely different from those related to fertilizers [4]. They are supplied to plants at very low dosage in order to induce beneficial effects, thereby they cannot provide any nutritional substance to plants directly [5,6,7]. Rather, they stimulate the capacity of plants to better acquire nutrients and use them for primary and secondary metabolism, and biomass production. They also aid plants to overcome stress conditions by eliciting the upregulation of enzymatic and non-enzymatic antioxidant systems [8,9,10,11].

HS are likely the most studied category of biostimulants and, even though many aspects of how they interact with plants are not completely known and need further investigation, the primary targets of their action have been undoubtedly identified. In the following sections we aim to describe (i) the structure of HS as defined by different analytical approaches; (ii) the role of HS at the root-rhizosphere zone; (iii) the effects of HS on plant growth and nutrition and the established or hypothesized mechanisms explaining their mode of action by complementing the dated literature with novel studies.

## 2. Structure of Humic Substances

While studying complex molecules, the first analytical approach is generally aimed to identify their molecular composition and, if necessary, the sequences of the individual components and which type of chemical bonds is implied. However, this method is not applicable to HS, whose bonds are more difficult to break down and the structural units are highly diversified and do not assemble in a regular sequence as in the typical bio-macromolecules (e.g., proteins, nucleic acids).

So far, the study of HS composition has been carried out under the action of strong oxidants (alkaline solution) or heat to determine the single structural units [12,13,14]. Nevertheless, the reactions obtained with either alkaline extraction or heat are extremely reactive, leading to the production of many artifacts that make the molecular recognition process further complicated. For this reason, linking the degradation products to their parent compounds is a very intricate issue.

**Figure 1:**
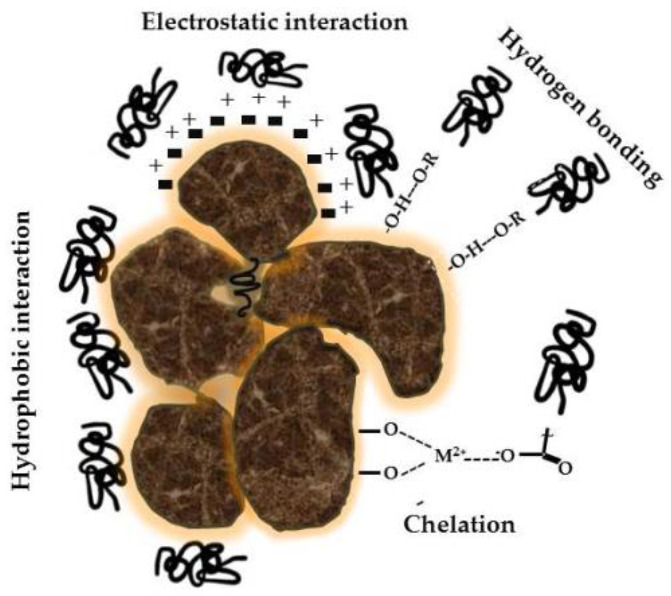
Associations between mineral colloids and humic substances are characterized by a variety of interactions and chemical bonds that make these structures stable in soils.

Alkaline extraction, first used by Achard [15], remains; however, the most common method for detecting the solubility of HS from soil, according to the International Humic Substances Society (IHSS) [16]. This type of extraction ensures maximal yields of organic material, since most of the organic matter is intimately bound to mineral colloids (Figure 1). Other extraction procedures using organic solvents [17] do not provide similar efficiency because the associations between mineral and organic colloids are of high structural complexity and binding strength [18,19].

In the early 21st century, a few researchers began to dismiss the terminology associated with HS [20] and renamed humic substances as the fraction of organic matter that remains structurally unknown [21,22,23]. This was due to concerns about the effectiveness of alkaline extraction and the chemical alterations caused by this procedure on the HS structure [24]. In addition, some studies rejected the hypothesis of any apparent relationship between the biological function of soil organic material and its alkali-extracted fractions by postulating that alkali extracts do not exhibit the same properties that they acquire during the humification process [21,22]. Conversely, comparing the 13 C NMR spectra of the extracted material with those of the native soil substances, Weber et al. [25] concluded that alkaline extraction does not alter the HS structure. Also, the same classes of substances were detected in a soil and in its derived-humic fractions and humin using pyrolysis-field ionization mass spectroscopy [18].

The debate is still ongoing, as highlighted by several remarks in response to these criticisms [19,26,27]. In this context, the sticking point is the absence of any chemical structure to be used as a reference control [28].

The HS elemental composition has been extensively studied and well documented [16,17]. Briefly, the content of various elements (C, H, N, O) of the IHSS standard and reference fulvic and humic acids ranged as follows: C from 50 to 60%, N from 0.7 to 5.1%, H from 3.5 to 4.8%, and O from 31.6 to 45.5% [29]. Overall, the average elemental composition of HS from various sources is reasonably consistent in the literature [17,30,31].

**Figure 2:**
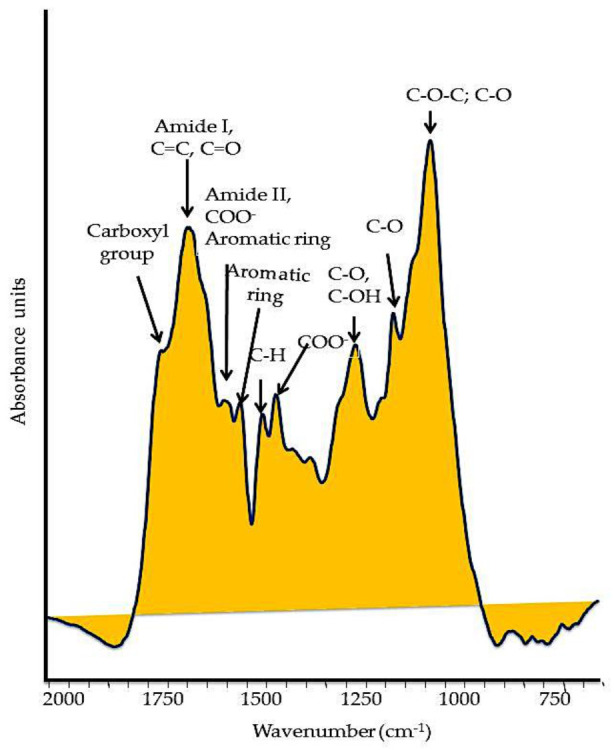
Typical FT–IR spectrum of a soil humic substance. The main oxygenated functional groups are reported in the spectrum.

Typically, HS bear functional groups (Figure 2) that contain oxygen (O), primarily in carbonyl (−C=O), carboxyl (C(=O)OH) attached to an R group, and hydroxyl (-OH) groups in alcohols and phenols; nitrogen (N) sets in functional groups of amines and amides, while sulfur (S−) in sulfhydryl groups. The various functions of HS are specially allocated to the carboxylic and phenolic groups, which are responsible for the weak acidity properties [29]. The concentrations of carboxyl and phenolic groups are commonly determined by direct titration, and usually range from 3.8 to 6.7 mmol g^−1^ and from 1.0 to 2.2 mmol g^−1^, respectively [29]. The pKa of most acidic groups ranges from 5 to 7.

The elemental composition, and consequently the functional groups, are strongly influenced by the pedo-climatic conditions [17], as well as by anthropogenic activities. In a comparative study by Plaza and Senesi [32], HS fractions extracted from soils that received different organic fertilizers (animal manures, composts, sewage sludge, and olive oil mill wastewaters) exhibited elemental compositions, E4/E6 ratio, fluorescence spectra, FT-IR spectra, ^13^C NMR spectra, organic free radical concentrations, intermediate between each amendment and native HS fractions from untreated soils. An indication of these results is that the HS fractions were susceptible to recent soil management with organic fertilizers. Recently, Pospíšilová et al. [33] studied the effect of biochar, compost, and digestate on HS structure. The authors concluded that the structural modifications detected in soil HS depended on the chemical characteristics of the amending materials. The electron paramagnetic resonance (EPR) spectra revealed that the fertilization with different organic materials led to changes in the HS magnetic properties due to the variable concentration and structure of radicals, while FT-IR spectra identified structural differences in HS mostly related to aliphatic and aromatic groups.

The study carried out by Hatcher et al. [34] using 13C-NMR showed that 35–40% of the humic structures is made up of single ring aromatic units. The authors assumed that fused aromatic structures are a trivial component of humic substances. Moreover, by examining the HS spectra, other C functional groups could be recognized that are associated with distinct molecular structures: alkyl C (aliphatic hydrocarbons, lipids), O-alkyl C (sugar-like), and carboxyl (peptide-like and organic acids).

The carbon-14 analysis of different humic fractions extracted from the same soil revealed that the older fractions gained higher proportions of aromatic and carboxylic C [35]. A similar process can be observed during the time sequence of coalification from peat to lignite and up to hard coals. During the coalification process, there is loss of moisture, volatile compounds, and consequently the concentration of C and aromatic macromolecules increases [25,36].

Recently, in agreement with elemental analysis, quantitative solid-state 13C NMR spectra has demonstrated that HS standards by IHSS contain a large fraction (28% and 33%) of polycondensed rings not bound to H or O and, oxygen-bonded non-protonated carbons, such as aryl ketone. Other constituents like C in alkyl, and -COOH groups are additionally present [37].

The aromatic nature of HS can be deemed as an indicator of stability against chemical and biological degradation [9]. In particular, the stability of HS seems to be associated with the formation of a complex and heterogeneous molecular network providing certain recalcitrance. In this context, the HS stabilization also occurs by adsorption of functional groups on clay mineral surfaces and through physical protection, within the pores of soil clay particles resulting in limited accessibility of microbes and enzymes [38,39]. Thus, a deeper comprehension of organo-mineral interactions is importantly advisable, since it may yield new approaches for soil carbon sequestration through HS stabilization.

**Figure 3:**
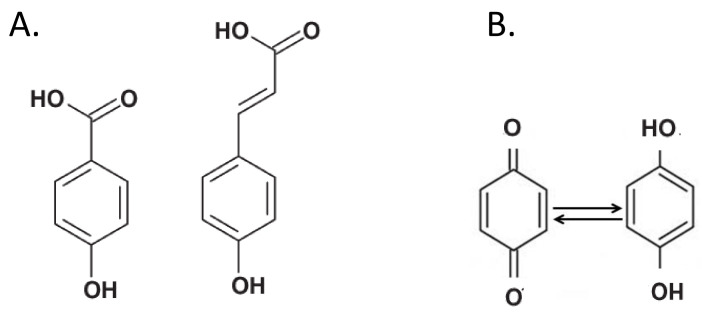
(**A**) Typical chemical structure of phenolic acids. These compounds are considered major components of soil humic substances. (**B**) Quinones (left) are groups that accept electrons and are reduced to hydroquinones (right).

Phenolic compounds have traditionally been considered as the main “building blocks” of humic substances [17]. In particular, phenolic acids, i.e., chemical compounds with an aromatic core and phenolic and carboxylic functions (Figure 3A), are valued at up to 35% in HS [40].

Research on HS has confirmed the role of dihydroxyaromatic acids as structural building blocks functioning in metal complexation [30,40,41].

A characteristic property of phenols is related to their reducing capacities or electron-donating capacities (EDCs) [30,39,42,43]. In a recent research, the EDCs of HS were investigated by electrospray ionization (ESI) coupled with Fourier transform ion cyclotron resonance mass spectrometry (FT-ICR-MS), total phenolic content and mediated electrochemical oxidation (MEO) analysis [44]. A strong linear correlation was found between EDCs, total phenols, and the proportion of polyphenolic formulas by ESI-FT-ICR-MS, containing medium oxygen content (0.4 ≤ O/C ≤ 0.67). The study confirmed that the major electron-donating capacities were due to the presence of phenolics, particularly polyphenolic compounds.

Quinones are electron-accepting groups of phenol origin that are first reduced to semiquinones, and then to hydroquinones (Figure 3B), i.e., compounds of higher stability [43]. The quinones can perform a redox cycle with their semi-quinone radicals and cause the formation of reactive oxygen species (ROS). The semiquinone-type free radical concentration (SFRC) in humus was used to assess the soil C stability. The SFRC was estimated by electron spin resonance (ESR) spectroscopy and correlated with indexes estimated by ultraviolet-visible (E4/E6), fluorescence intensity (FI), 13C NMR and FT-IR spectroscopies of HS [45,46]. More recently, the low-molecular-weight HS fractions were found to exhibit great reducing capacity due to the presence of a large amount of quinones [47].

The structure of HS is operationally defined in (i) humic acids (HA), which are the fraction soluble in alkali, but insoluble during subsequent acidification, and (ii) fulvic acids (FA), which are soluble in both alkali and acids [17]. At alkaline pH, phenolic and carboxyl groups are extensively deprotonated, and the repulsion forces favor the dispersion of HS because intramolecular hydrogen bonds are completely disrupted [48,49,50]. Rheological results confirmed the extending configuration of the HS at alkaline pH and its ability to increase repulsive forces in suspension, promoting their dispersion [51]. As the pH decreases, functional groups are protonated and repulsion effects decrease, driving the molecule to dispose on a coiled structure, which is followed by intermolecular aggregation [18,30,31,48,49,50]. The coiled configuration leads to complete expulsion of the water molecules surrounding the HS surface and, as a consequence, the HS becomes insoluble and precipitate [18,30,31,51]. This effect is also observed by treating HS with weak acids, which cause the decrease of HS apparent molecular size or disintegration as weak non-covalent interactions, such as van der Waals, π-π, and CH-π, are disrupted [48,52]. Such processes may be mimicking the activity of root exudates, containing low molecular weight organic acids, and influence the molecular size and solubility of HS in the soil [53,54]. In addition, the carboxyl groups also contribute to determining HS solubility and biological reactivity [55,56].

## 3. Relationship between Structure and Biological Activity

The complexity of HS structures and their related bioactivity in plants has been largely described [1,2,9,57]. Although several approaches have been used in studies concerning this topic, so far a direct relationship between chemical structure and effects on plant metabolism has not yet been fully clarified.

Initially, the molecular size, hydrophilicity and specific functional groups of HS attained great relevance [5,58]. Zancani et al. [59], identified the fraction III, the most hydrophilic and smaller in molecular size, among several humic acids. This fraction induced partial relief of Pi starvation by increasing the total amount of cellular phosphate, ATP and glucose-6-phosphate levels, as well as the activity of secreted acid phosphatases in tobacco BY-2 suspension cell cultures. In another study, a compost was subjected to sequential chemical fractionation and the resulting fractions were tested on maize seedlings to evaluate the effects on growth and nitrogen metabolism. The increased stimulatory activity involved fractions with structurally unbound molecules that appeared sufficiently hydrophilic or with a less complex structure to be more easily accessible to plants [60].

The role of different molecular size fractions of HS on root growth was also explored in *Arabidopsis thaliana* and maize seedlings [61,62]. Results indicated that by decreasing the molecular size of HS, the carbohydrate content and alkyl chain length decreased, as well as the aromatic carbon. Even though the induction of root growth in *A. thaliana* and maize seedlings was induced by all HS, the intensity of the effect depended on the HS molecular size and the plant species. A further investigation showed that the hydrophobicity index (HB/HI) of HS, obtained using NMR parameters, was correlated with the lateral root hair emergence, while the hydrophobic carbon content was negatively correlated with the induction of lateral roots [62].

Other research has confirmed the HS role in the induction of lateral root emergence of different plants [63,64]. However, the remaining unresolved issue is standardizing the structural, chemical properties of HS related to their bioactivity, which seems to be dependent on many variables like the origin, the extraction and purification procedures of HS. As an example, in some research the induction of lateral root emergence was positively correlated with the lignin-derived aromatic component, whereas negatively correlated with cellulose derivatives [63]. Conversely, simple phenolic compounds, such as hydroxy-substituted benzoic and cinnamic acid derivatives were considered the most active components in other studies [56,64].

A study testing 37 humic fractions—characterized by isotopic composition, structural characteristics, and chemical properties responsible for stimulation of root traits—showed that the fractions shared some structural similarity although differing in their stimulation activity on plant roots [8]. In this context, the lability (N, O aliphatic chains, and carboxyl group) and recalcitrance (unsubstituted aromatic and aliphatic structures) of HS were associated with the nature and strength of their biological activity on the root. In more detail, the labile part of the HS fraction induced the development of the root length and lateral root emergence at low concentrations, while the more recalcitrant fraction influenced root growth at high concentrations.

Differences in bioactivity of HS extracted with either water (HLAw) or the IHSS’s recommended method (HLA), indicate that the extraction procedure might influence the induction process of lateral roots emission. The HALw activity was related to aliphatic and oxygenated compounds, while the stimulation of root growth was attributed to the aromatic compounds in HLA. Higher concentration of HLAw was therefore required to elicit similar stimulatory effects as HLA. Although the two fractions have a similar composition, differences between the main structures are likely to exert a different impact on the root [65].

The auxin-like activity of humic acids responsible for root changes has been largely investigated [2,66,67] and will be described in detail in a further section of this review. HS from vermicomposting, produced using leather waste and cattle dung at different stages of maturity, differed in auxin-like activity. Overall, auxin-like activity increased as the vermicomposting process progressed. The molecules implicated in auxin-like activity were identified to be carboxylic acids and amino acids [68,69].

## 4. Soil–Root Crosstalk

The term soil-root crosstalk was introduced by Nardi et al. [67] and Urrutia et al. [70] to describe the relationship between plant biological activity and rhizosphere soil.

**Figure 4:**
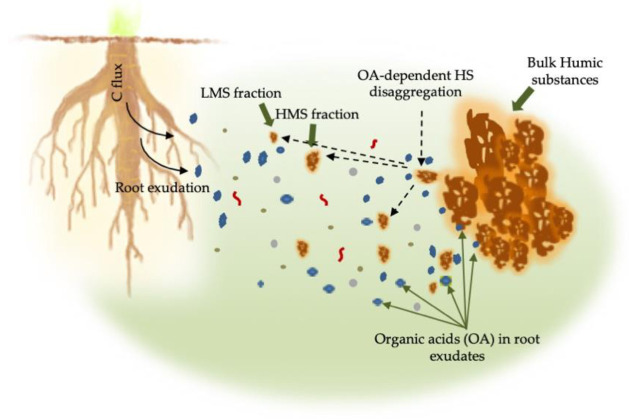
Root exudates contain substances, including low molecular weight organic acids (OA) that may influence the solubility of soil HS (bulk HS) by inducing their disaggregation to produce LMS and HMS fractions.

The root biological activity consists in the release of root exudates and secretion of root border cell into the rhizosphere. Substances in root exudates are ions, low and high molecular weight compounds able to modify the soil properties. Among them, organic acids have long been recognized as key factors in soil formation and evolution. Organic acids are capable to alter the mineral weathering conditions by changing the soil complexing capacity, the pH, and the amount of mineral elements [71]. They can alter the macrostructure of the HS promoting the release of small fractions [61,66] (Figure 4). These fractions can target the cell receptors at the surface of the root or enter the root cells and induce biological activity.

Nardi et al. [72,73,74] demonstrated through in vitro experiments that organic acids (e.g., fumaric and succinic acids) from maize exudates were able to modify the molecular size of HS, shifting them from high to low molecular weight.

Exudates from crops and forest species have also been used to extract HS from the soils, thereby proving that exudates are more efficient in recovering low molecular size (LMSs) biologically active substances than traditional alkaline solutions [53]. The LMS obtained from these exudates showed differences in C, N, and organic acid contents, as evident in Table 1.

**Figure 5:**
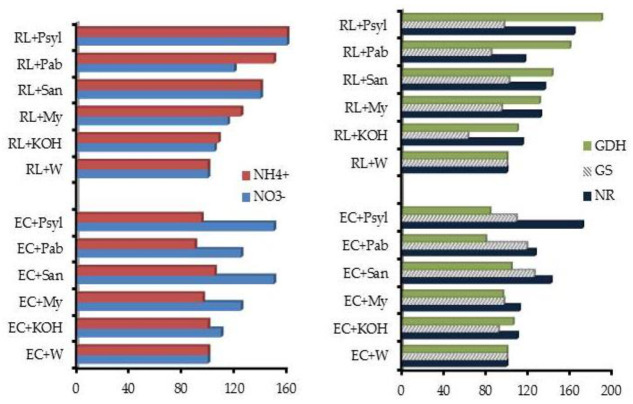
NO_3_^−^ and NH_4_^+^ uptake (**left**) and nitrate reductase (NR), glutamine synthetase (GS) and glutamate dehydrogenase (GDH) activities (**right**) in *P*. *sylvestris* seedlings treated with low-molecular-size organic fraction extracted from Eutric Cambisol, EC and Rendzic Leptosol, RL by maize (cultivar Mytos and Sandek) or forest root exudates [73]. P. syl = *P.* *sylvestris;* P. ab = *P. abies; San =* Sandek¸ My = Mytos; W = water.

The bioactivity of low molecular size (LMS) HS was assessed in *P. sylvestris* seedlings by determining the hormone like activity, nitrate uptake and nitrogen metabolism [73]. The results demonstrated that the chemical composition of LMS fractions and soil type are important predictors in modifying nitrogen metabolism (Figure 5), and pinpointed the importance of root exudates in the separation of the plant’s active biological components endowed with hormone-like activity.

The molecular weight dynamics and change in bioactivity of HS in the presence of organic acids along with their profile were also investigated by Canellas et al. [46] in maize roots. Results from this study indicated that HS effects on plant growth were dependent on variation in organic acid root exudation profile and associated changes in HS structure and assembly.

Nardi et al. [2] therefore postulated that HS may act as signaling molecules in the rhizosphere releasing hormones and hormone-like substances or promoting their production at plant/microorganism level. On the other side, humic fractions can interact with plants, which results in alteration of plant metabolism and the release of molecules into the rhizosphere, thus influencing the specific crosstalk between organic and humic matter in the soil.

Very recently, Baia et al. [74] demonstrated that the addition of organic acids typically found in the rhizosphere increased drought tolerance in crops (maize, rice, and wheat) by eliciting the activation of the jasmonate (JA)-signaling pathway. In addition, HS at a suitable concentration, can act as a chemical triggering agent that leads to plant acclimatization and increased tolerance to abiotic stress.

## 5. Biological Activity of Humic Substances in Plants Defines Their Role as Plant Growth Promoters

### 5.1. Effects of Humic Substances on Plant Growth via Hormone-Signaling Control

Owing to their chemical properties and molecular structure, HS are unequivocally recognized as plant growth promoters. In many studies, the application of HS was effective in promoting plant growth within a short period since applied, giving evidence of their elevated biological activity [75,76,77]. The effect of HS on plant growth toughly depends on the source, dose, content in bioactive molecules and molecular weight of the humic fraction, mode of HS application. While the source is relevant in shaping the abundance and type of active functional groups and the structural properties of HS [53,76], the dose applied and the concentration of specific bioactive molecules is particularly critical for maximizing the positive effects on plant growth, which generally follows a bell shape trend in response to increasing HS dosage [61,63,69,76,78]. Indeed, the HS application rate nonlinearly impacts on the plant growth response, as the canonical dose–response curve displays positive correlation of root growth with increasing HS concentrations, followed by a decline of growth at high HS concentrations [76,78,79]. Shoots display a similar trend as roots in response to HS, but the magnitude of the response could be lower [78]. However, Rose et al. [78] in their meta-analysis of plant-growth responses to HS, appraised comparable shoot and root dry weight increases in response to HS application, which accounted for 22 ± 4% and 21 ± 6%, respectively. They concluded that the actual variation in magnitude of shoot and root growth in response to HS is affected by all those factors mentioned above, especially considering that multiple chemical functional groups of HS, might behave differently under different environmental conditions, or when applied to different plant species.

Once applied, the mode through which HS interact with plants differ based on their size: the low molecular size (LMS < 3500 Da) fraction can easily enter the root cells, while the high molecular size fraction (HMS > 3500 Da) is hardly absorbed and generally interacts with the cell wall components and the root membrane receptors to trigger internal signal transduction cascades [66,76,80,81,82]. Recently, the intensity of the stimulatory effect of HS on plant growth as a function of the dose and the molecular size of the HS fraction applied was determined in garlic plants [76]. The authors found the maximal effect triggered by HS was dependent on both the dosage and the HS size.

**Figure 6:**
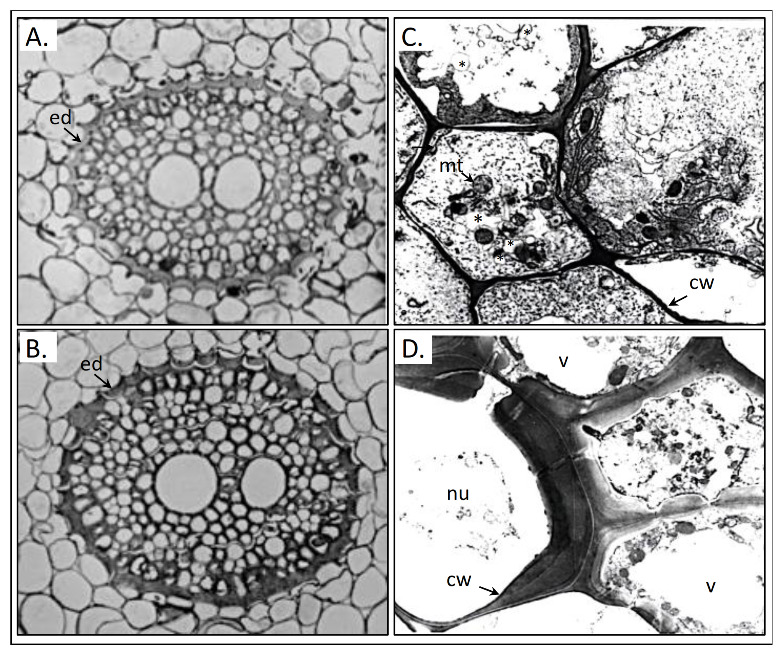
Central cylinder of wheat roots untreated (**A**) and treated (**B**) with HS. Xylem vessels of untreated roots exhibit lower differentiation degree and thinner cell walls compared to HS-treated roots. TEM micrograph of wheat roots untreated (**C**) or treated (**D**) with HS. In the treated samples, root cells have cell wall (cw) with high thickness. ed: endodermis; mt: mitochondria; nu: nucleus; v: valcuole; *: provacuoles.

The immediate primary target of HS is the root, which manifests increased elongation and early differentiation processes at the root tip and primary zone [76,83]. Depending on the HS fraction, early differentiation patters might develop either in the central cylinder, with relevant impact on water conductivity and nutrient flux intensity, or in the cortex, leading to the increase of root diameter and resource storage [76]. Details about the effect of HS at the cellular level in roots are depicted in Figure 6.

**Figure 7:**
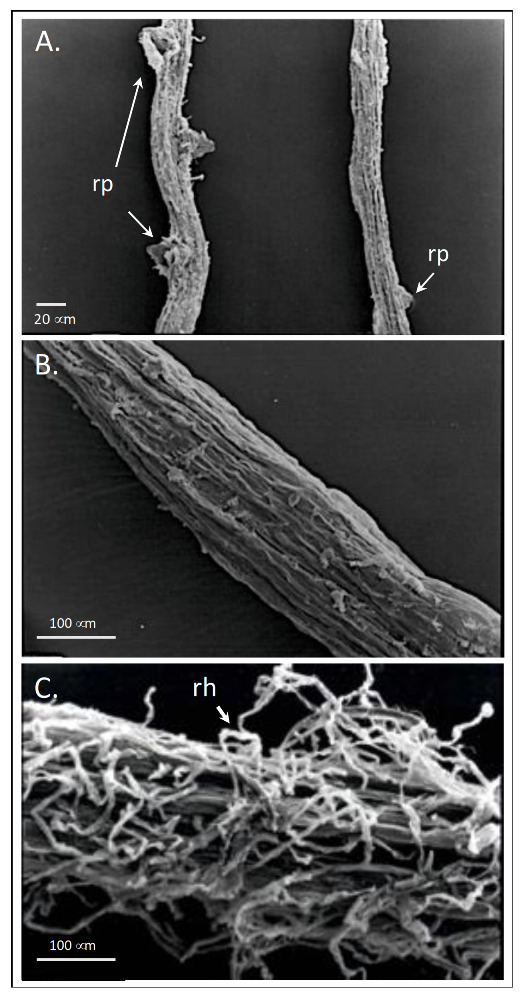
SEM micrographs of the 0–20 mm region behind the root tips of wheat seedlings surface. Plants were grown in Hoagland solution and treated for the last 2 days with HS. (**A**) plant treated with HS on the left, untreated plant on the right; (**B**) = untreated plant; (**C**) = plant treated with HS. rp: root primordia; rh: root hair.

**Figure 8:**
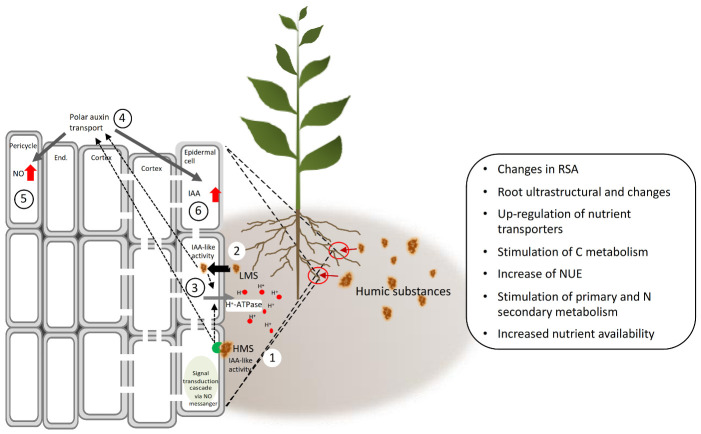
Mechanism of action of HS at root level to induce lateral root emergence and stimulate root hair density and length, and metabolic-physiological targets in plants. HMS interact with membrane receptors to induce cascade signaling inside root cells (**1**); LMS enters the root cells (**2**). Both HMS and LMS stimulate the activity of the root membrane H^+^-ATPase (**3**); and the auxin polar flux (**4**) to promote auxin and NO accumulation at the pericycle cells to enhance lateral root emergence (**5**). HMS and LMS induce accumulation of auxin in the root epidermal cells, resulting in increased root hair formation and cell elongation (**6**).

Beside root ultrastructure modifications, HS can induce lateral root emergence, root elongation and root hair [81,84,85,86,87] (Figure 7). The mechanism implied, is apparently mediated by HS-dependent effects on auxin polar transport and nitric oxide (NO) signaling pathway [31,66,82,86] (Figure 8). The role of auxin in modulating root growth and morphology in this case lies in the fact that HS enclose in their structure indole-3-acetic acid (IAA) and other molecules (e.g., phenylacetic acid, indole-3-butyric acid, carboxylic acids, amino acids) endowed with IAA-like activity [68,69,80,84]. These phytoregulators could be of either of microbial or plant origin in soil, and their contents vary depending on the soil type. Overall, they are more abundant in the rhizosphere than in the bulk soil, likely because of greater microbial biomass and activity and root exudation activity [88]. HS also contain aromatic groups of high biological activity, especially phenol-C groups, which account for part of their IAA-like activity [89,90]. Zandonadi et al. [82] postulated that HS and auxins trigger root development by exploiting mechanisms that make use of NO as a messenger, whose accumulation at specific sites of root emergence—i.e., pericycle cells—is critical in the early stages of lateral root development (Figure 7). In support of this hypothesis, applying inhibitors of auxin-signaling (PCIB) and efflux (TIBA) did not hamper the effect of NO, which was however decreased by NO scavengers. NO is generated through two main metabolic routes, one enzymatic involving the enzymes nitrate reductase [91] and a putative nitric oxide synthase [92], and a one non-enzymatic that uses NO_2_^−^ as a precursor and takes place on the plant cell surface in response to apoplast acidification [93,94]. The increase of nitrate reductase activity was observed by Vaccaro et al. [60] and Vujinović et al. [77] in maize plants treated with HS, which might indicate higher NO production along with increased N assimilation.

The effect of HS on NO signaling has been also associated with the increased activity of the root plasma membrane (PM) H^+^-ATPase, as observed in the case of exogenous IAA application to in maize and cucumber plants [31,82]. Thus, HS are believed to behave as exogenous auxins and regulate root growth and morphology by targeting cross induction of root plasma membrane (PM) H^+^-ATPase and NO generation. In addition to the root PM H^+^-ATPase, vacuolar H^+^-ATPases and H^+^-pyro-phosphatase are similarly activated by HS treatment [81]. This is because the auxin-like induction of protein synthesis and activation of the plasma membrane H^+^-ATPase in roots can be associated with the acid growth theory [95] based on IAA-induced cell wall loosening, to which tonoplast H^+^ pumps and the membrane-bound pyrophosphatase (H^+^-PPase) extensively participate [84]. These pumps indeed contribute to the preservation of the H^+^ electrochemical gradient that is needed to guarantee the osmotic pressure of the vacuole high enough to allow water uptake and turgor maintenance. The activity of the root plasma membrane H^+^-ATPase seems to be crucial also for the increase in shoot growth mediated by HS [31], as its inhibition resulted in failure of HS-dependent shoot growth induction. HS applied at the root area can induce physiological responses in the shoot, that are regulated by established targets of auxin action, like the stomatal opening regulator phospholipase A2 [68].

More recently, the positive effect of HS on root elongation in *Betula pendula* Roth and *Alnus glutinosa* L. Gaertn was associated with the HS-dependent modulation of the auxin polar transport, as revealed by the increased expression of transcripts for the ABCB transporters ABCB1 and ABCB19 [86].

Although several studies so far definitely confirm the hormone like-activity of HS, this property has long been under debate [85]. Initially, only indirect evidence was inferred about the content of IAA in HS being sufficient to be biologically stable and active [58]. Nevertheless, the occurrence of IAA in HS has been further corroborated in a plethora of studies using immunological detection or spectrometric identification approaches [76,80,84]. The second step was to verify that IAA enclosed in HS was really responsible for the auxin-like activity of HS, thus affecting root morphology and growth, and altering specific targets of IAA action in plants. A series of works focused on this topic. The LMS fraction and IAA were found to induce similar morphogenetic effects in *Nicotiana plumbaginifolia* leaf explants likely via a stimulatory effect on the growth-marker enzymes peroxidase and esterase [96]. Conversely, in homogeneous carrot (*Daucus carota* L.) cell cultures, the LMS fraction rich in carboxylic groups and IAA were shown to bind the carrot cell membranes in the same manner. Increased activity of peroxidase and esterase was repeatedly reported in plants receiving HS [76]. Further evidence of a direct role of HS in plant physiology was provided by Zandonadi et al. [81] and Trevisan et al. [97], who postulated that HS and IAA shared common effects on root growth by inducing the lateral root proliferation, and by Canellas et al. [84] who identified the PM H^+^-ATPase as a major target of IAA in HS. To complete this framework, Dobbs et al. [98] concluded that HS biological activity requires the activation of the auxin transduction pathway in maize and tomato plants, and hypothesized that HS can behave as a sort of ‘buffer’ by absorbing or releasing hormone-like signaling molecules, depending on changes in the rhizosphere properties driven by the activity of PM H^+^-ATPase and organic acid exudation. This was because HS could not induce lateral root formation in a tomato mutant (dgt) defective for auxin response.

Recent studies postulate that further compounds other than IAA might control root developmental programs in plants treated with HS by eliciting endogenous signals, especially because hormonal-associated effects do not always correlate with the IAA content in HS, and auxin-responsive responsive genes, such as IAA5 and IAA19, are not consistently modified by HS [75,97].

Controversial findings concerning the IAA-like activity of HS were, however, reported by Schmidt at al. [85]. If water-soluble humic molecules were able to stimulate the proliferation and elongation of root hairs in wild type plants, which is as typical response to increasing auxin concentration at the root epidermal cells, they could not conversely complement the phenotype of *Arabidopsis* auxin-mutants low in root hair number. Also, unlike IAA and ethylene, they could not restore the normal root hair development in mutants defective in root hair initiation. The authors concluded that HS cannot replace IAA (and ethylene) in the plant response associate with root hair formation, and that HS may affect the root shape and growth without any substantial effect on auxin signaling. This theory, however, appears speculative because it is not endorsed by the chemical and spectroscopic characterization of the HS applied, neither by the analysis of their IAA content, which is mandatory to explain the effects of HS in plants. In addition, the extraction procedure does not allow comparison with existing literature on this topic.

Along with the hormone-like activity of HS, a number of studies have reported the increase of IAA, NO, ethylene, and ABA contents in roots of plants treated with HS [31,84]. The crosstalk between auxin and ethylene in lateral root proliferation has been extensively documented, while the increase of ABA along with auxin was reported to be crucial in determining changes in root growth of cucumber plants applied with HS at the root area [31,84]. The use of inhibitors of IAA biosynthesis did not allow secondary root development in these plants, but HS maintained their capacity to induce the increase in total root biomass [83]. However, this effect of HS was no longer evident in plants treated with inhibitors of ABA biosynthesis [31].

Most of the effects reported for HS on plant growth derive from studies where HS were applied at the root area, either in hydroponics or to the soil. However, other studies have highlighted the capacity of HS to promote plant growth when used as foliar sprays [1,75,84]. In this case, the mode of action of HS appears to involve unique plant nutritional, metabolic and physiological responses [75]. Foliar-applied HS promoted both shoot and root growth of cucumber plants, as well as root volume and primary root elongation, but reduced lateral root emergence [75]. This finding suggested that HS likely activated a long-distance control of the root system architecture (RSA) inducing substantial changes in the primary root traits. Nevertheless, foliar-applied HS increased auxin (indoleacetic acid, IAA), but not abscisic acid (ABA) in roots, and did not stimulate the activity of the root H^+^-ATPase, neither nutrient accumulation in the shoot [75]. As mentioned fore above, auxins are important determinants in lateral root development [83], while ABA has a potential positive role on the whole root dry biomass [31]. Possibly, foliar-applied HS may elicit further regulatory factors and complex signaling hormonal pathways that affect RSA by opposing to the canonical action of auxin, thus explaining the failure in lateral root emergence, and by complementing ABA effects for root dry biomass. The decrease of ABA in cucumber plants could be though important for shoot growth, which is known to be impaired by high ABA concentrations [99]. The null effect of foliar-applied HS on the H^+^-ATPase activity in cucumber plants indicated that this protein is a major target of HS action only whether HS are applied at the root level, and that H^+^-ATPase was not responsible for the increase of cytokinins observed in the plants [75].

### 5.2. Humic Substances Promote Plant Growth by Enhancing Nutrient Availability, Acquisition, and Use Efficiency

Similarly to other classes of biostimulants, HS act as plant growth promoters by exerting direct and indirect effects on plant nutrition [2,100]. Beyond modifications of the root anatomy and system architecture traits optimized for better soil exploration and nutrient interception, HS can influence other nutrient acquisition strategies of plants, for instance by modulating the expression of transporters involved in nutrient primary uptake, increasing organic acid root exudation, and favoring the plant interactions with beneficial rhizosphere microorganisms, also termed plant growth promoting bacteria (PGPB) [2,46,101,102]. Recent studies have also evidenced the capacity of HS to ameliorate plant growth by enhancing their root hydraulic conductivity, an effect dependent on the structural conformation assumed by HS in solution [31].

Direct effects of HS on plant nutrition include the promotion of nutrient uptake by plants, addressing specific nutrient master regulators and nonspecific targets, especially at the plant cell membrane [102,103], while indirect effects of HS are those related to the soil environment, whose chemical, physical and biological properties are generally amended by HS [2]. In the latter case, HS applied to soil were proved to enhance the stability of soil aggregates [20,104], thus reducing soil erosion and preventing C and N losses by leaching. Furthermore, functional groups of HS exhibit high affinity for inorganic and organic ions and a number of molecules that reside in soil [105], and can form complexes with metallic cations—like Zn, Mn, Cu, Fe, as well as with inorganic P—by protecting them for leaching losses and maintaining them available for plant uptake. The formation of such complexes is possible because of the occurrence of oxygen-, nitrogen-, and sulfur-containing functional groups in the HS structure [106], and is particularly important because nutrient deficiency associated with low availability of such nutrients as Zn, Fe, and P is a widespread issue in agriculture.

The action of HS on ion uptake has received mounting attention in the last decades and many studies revealed that HS-induced amelioration of nutrient acquisition by plants is dependent on multiple factors, primarily the HS origin, type, dosage, and structural properties, the pH of the rooting medium, the exogenous nutrient concentration and the plant species [2,66]. Potentially, HS might increase the acquisition efficiency of all nutrients, due to their capacity to modulate the activity of the root PM H^+^-ATPase, which is a recognized as a marker of biostimulant action and contributes to regulate the rhizosphere pH, thereby affecting nutrient availability [107]. Nevertheless, so far most research has evaluated the effect of HS in improving the capacity of plants to acquire N, P, and Fe, for which deficiency in agroecosystems is a more relevant issue and poses environmental concerns [106]. In the case of N and P, HS were proved to upregulate the expression of nitrate and phosphate transporters, respectively [108,109]. Enhanced expression of N transporters (e.g., NRT1.1 and NRT2.1) likely accounts for increased nitrate uptake reported in a number of studies conducted in maize, wheat and oil seed rape [53,108]. Increased nitrate uptake by HS has been associated with enhanced root to shoot mobility of the hormones cytokinins (CK), with positive outcomes in plants in terms of leaf/shoot growth [83] and protection of the photosynthetic machinery under stress condition [9].

With respect to P, upregulation of the root high-affinity Pi transporter gene LePT2 was observed in tomato plants treated with HS, regardless of low or high P supply [109]. Furthermore, HS altered the distribution of P species in the leaves of these plants as revealed by ^31^P-NMR, by promoting the accumulation of Pi at high P, while glycerophosphodiester and phosphorylcholine at low P. These changes in P forms apparently indicate a role for HS in plant adaptation to low P input.

Recently, Zanin et al. [106] have elegantly reviewed the role of HS in Fe nutrition and pointed out that soluble Fe-HS complexes represent an available source of Fe for plants, but can also significantly affect the plant physiology. The formation of such soluble complexes is mediated by carboxylic and phenolic groups and aliphatic domains of HS [17,30,37,40,41], but HMS can also stabilize amorphous Fe oxides by forming insoluble co-precipitated that represent a long-term Fe stock in soil potentially available for plant uptake [110]. The capacity of HS to make complexes with Fe might have an impact on P nutrition by increasing P availability, because P can be linked to HS via Fe-bridge [70,111]. On this account, the commercial substitution of commercial Fe-chelates proved to be effective in promoting Fe uptake and being economically valuable [112].

The effects of HS or Fe-HS complex application to plants have been studied at different levels and many physiological and molecular responses associated with Fe deficiency are described [112,113] and are apparently influenced by the nature of the chelating agent [114]. Aguirre et al. [112], in particular, investigated the expression of Fe-related genes in cucumber plants in response to HS and found that those genes were variably and transiently regulated. Specifically, the gene coding for the root PM H^+^-ATPase isoform CsHA2 was upregulated by HS, but not the gene coding for the CsHA1 isoform, and the Fe (II) transporter Cs (IRT1) and Fe (III) chelate reductase CsFRO1 genes were upregulated within 72 h, but downregulated at 96 h. These effects at the transcriptional level concurred with the increase of the root H^+^-ATPase and Fe (III) chelate-reductase activities. In a more recent study by Tomasi et al. [113], the supply of Fe-WEHS complex to Fe-deficient tomato plants accounted for the upregulation of the root Fe (III)-chelate reductase (LeFRO1) and Fe transporter genes, LeIRT1 and LeIRT2. Such an effect was faster and more intense compared to the complexes Fe-citrate or Fe-phytosiderophores (PS). Similar results were reported by Billard et al. [115], who showed that HS isolated from black peat could trigger the upregulation of the IRT1 gene in roots and leaves of rapeseed plants, thus increasing their Fe content. Consistently, Zanin et al. [116] evidenced the upregulation of CsFRO1, CsIRT1, and CsNRAMP in leaves of Fe-starved cucumber plants treated with Fe-WEHS rather than with Fe-PS.

HS are reported to render some nutrients more available for the uptake by plants by promoting the activity of soil bacterial communities [101,117] and the establishment of plant root symbioses with beneficial rhizosphere microorganisms, likely because of the enhanced release of organic acids by HS-treated plants [101]. HS are moderately recalcitrant to bacterial degradation and HS-induced anatomical and morphological changes of roots may promote the establishment of a plant-microorganism mutualistic symbiosis by fostering the number of rhizosphere communities and chemotaxis, bacterial attachment and survival on the root surface, and endophytic colonization [102]. HS were previously reported to intensify the root exudation of organic acids into the rhizosphere of maize plants [118]. Carboxylates, beyond increasing the availability of poorly soluble nutrients, represent a precious C source for supporting the growth of symbiotic microorganisms. Also, they can modify the supramolecular arrangement of HS by releasing bioactive molecules that may affect plant growth and modulate root architecture, while increasing epiphytic and endophytic bacteria colonization. HS cause heterogeneity of the root surface, border cells formation and excretion from root tips, and possibly might modulate by the release of chemoattractant/antimicrobial compounds into the rhizosphere [102]. These traits can facilitate the anchoring of microorganisms to the root surface and their proliferation nearby the border cells [1]. Furthermore, it has been postulated that the stimulatory effect of HS on the root cell membrane H^+^-ATPase, resulting in increased activity of cell wall-associated hydrolases, would promote the bacteria entrance into the root in some cases [102].

In addition to promoting the nutrient acquisition strategies of plants and the availability of mineral elements in the rhizosphere, HS stimulate the nutrient translocation and nutrient use efficiency of crops by acting on primary and secondary metabolic routes [60,90,108,119]. In particular, N assimilation is definitely a key target of HS action in plants, as the activity and gene expression of major enzymes as nitrate reductase, glutamine synthetase, GS, and glutamine amino transferase, GOGAT, as well as the content of proteins has been reported to increase in plants after treatment with HS [60,90,119]. Energy-processes like cells respiration and photosynthesis are also promoted by HS, as revealed by the increase in glucose-6P-isomerase and pyruvate kinase [120] and Rubisco [119] activities. Enhanced cell respiration along with photosynthesis will ensure adequate C supply and energy inputs for energy-requiring processes, like those related to nutrient transport and biomass production. Under this condition, root exudation of C metabolites that mediate root-rhizosphere interactions is likely favored.

## 6. Conclusions

HS are essential components of soil organic matter, with manifold roles in the soil environment and in the plant–soil–microbial interactions. Their complexity is widely recognized and their singular molecular and structural properties are responsible for a plethora of effects in plants and regulate their responses to changing environment. HS are endorsed as positive growth regulators and the mode through which they act in plants is a matter of debate that has fascinated many scientists in the last decades. The capacity to behave as hormone-like substances is likely the most intriguing trait of HS. Nonetheless, we believe much remains to discover about them, and future research should be more focused on unravelling the complex association between molecular structure and biological function in order to enhance the efficiency of HS use in sustainable technologies in agriculture.

## Figures and Tables

**Table 1:** Dry weight of maize cultivars and forest seedlings and the composition of their root exudates used for soil extraction (modified from [53]).

	d.wt. (g)	pH	C (%)	N (%)	LMS (mg g^−1^ Root d.w.)	
					Fumarate	Succinate
*Paolo*	0.18	8.2	0.93	0.62	0.007	0.78
*Sandek*	0.23	6.2	1.36	0.37	0.034	5.55
*Picea abies*	0.01	6.3	0.02	0.02	n.d.	40.76
*Pinus sylvestris*	0.02	5.9	0.09	0.09	n.d.	84.09

d.wt. = dry weight; n.d. = not detected; LMW = low molecular size.

## Data Availability

Data Availabilite on request from the corresponding author.
